# Supplementary material for: Silencing of dentate gyrus inhibits mossy fiber sprouting and prevents epileptogenesis through NDR2 kinase in pentylenetetrazole kindling rat model of TLE
Source: PLoS One. 2023 Apr 12;18(4):e0284359. doi: 10.1371/journal.pone.0284359 (PMC10096303; doi:10.1371/journal.pone.0284359)
Supplement: S1 Fig — (PDF) [file pone.0284359.s002.pdf]

C: control rat　　A: DREADD+CNO10mg

LOAD ORDER:FROM left side to right side captured by EPSON V300.

A　A　C　C　C　C　C　　　　A　A　C　C　C　C　C

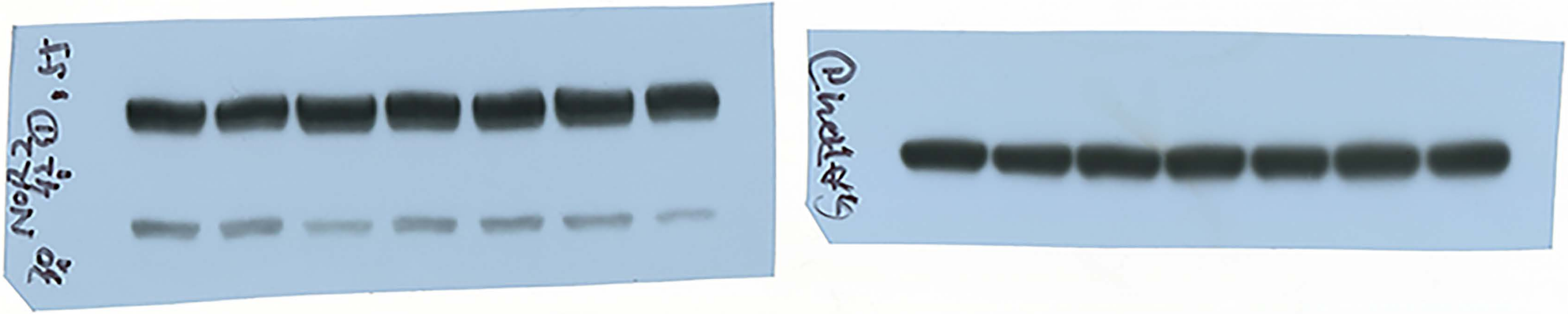

C:control rat   A：dreadd+cno10mg

panel of figure was generated from that original image

LOAD ORDER:FROM left side to right side captured by EPSON V300.

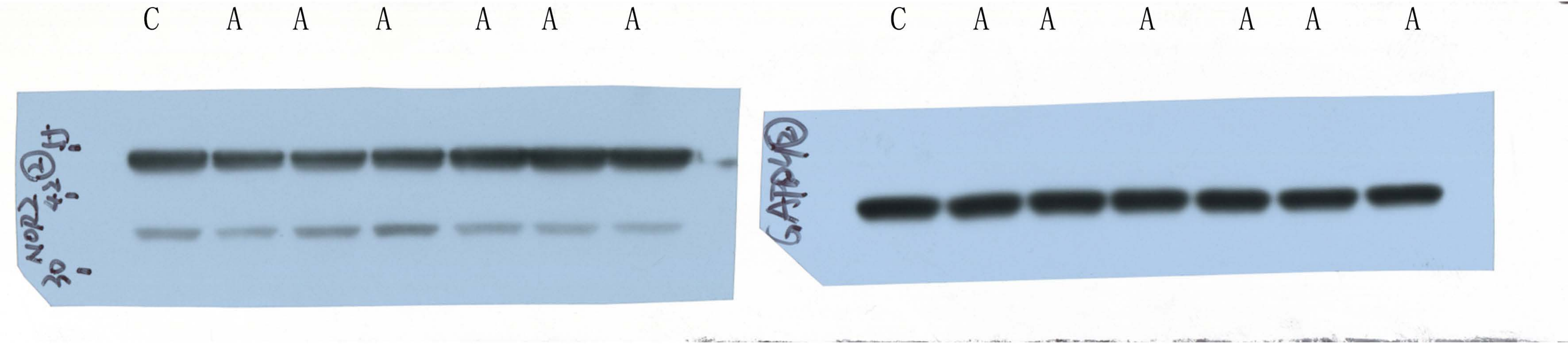

C： control rat　　A： DREADD+CNO10mg

LOAD ORDER:FROM left side to right side captured by EPSON V300.

C　　C　　A

55

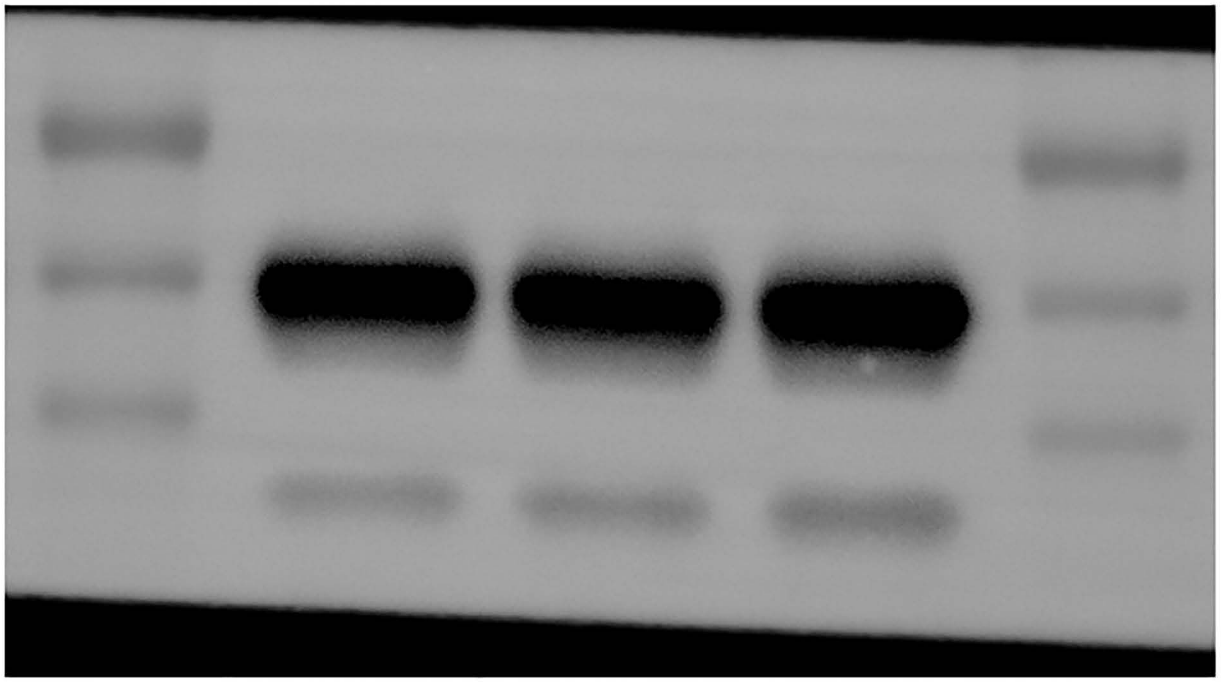

NDR2

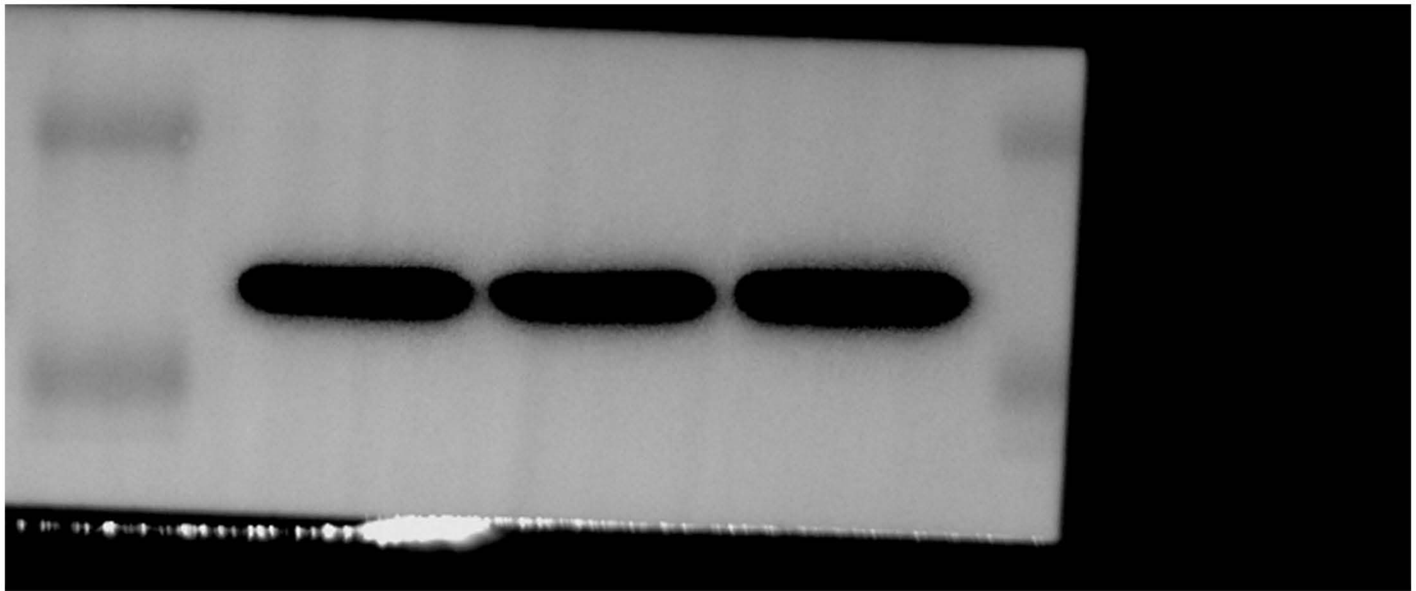

GAPDH

30

A: DREADD+CNO10mg B DREADD+CNO+NDR2

LOAD ORDER:FROM left side to right side captured by EPSON V300.

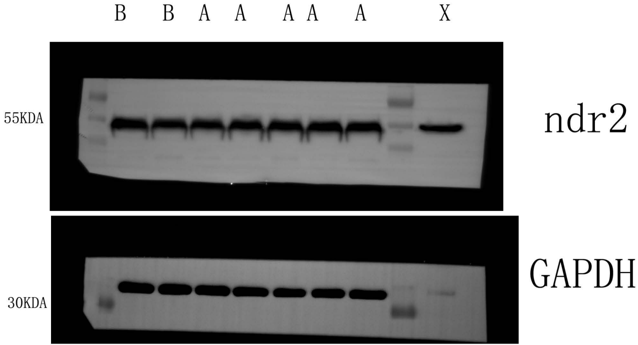

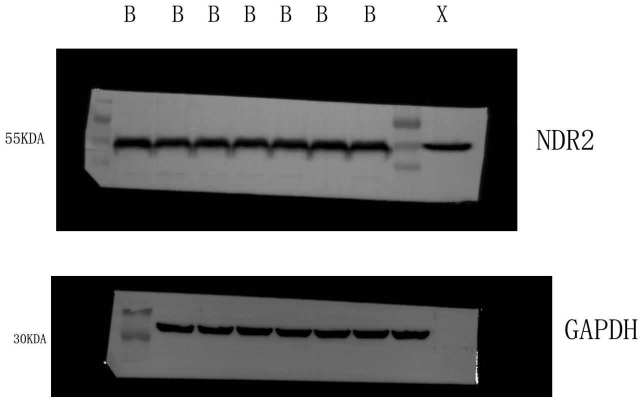

B B B B B B B X
55KDA
NDR2
30KDA
GAPDH

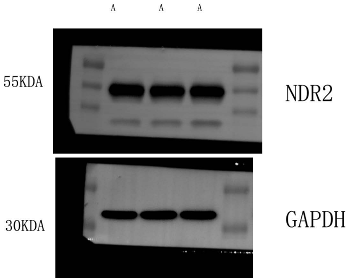
A
A
A
55KDA
NDR2
30KDA
GAPDH

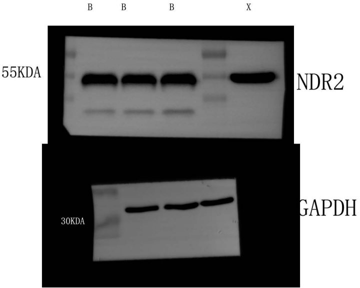
B
B
B
X
55KDA
NDR2
30KDA
GAPDH

A A A B

panel of figure 4B was from this original image

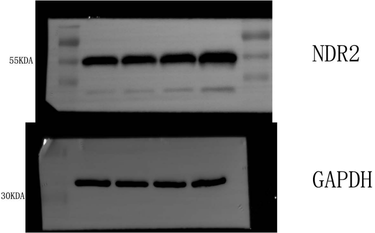

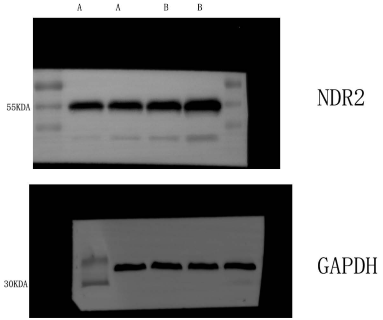
A
A
B
B
55KDA
NDR2
30KDA
GAPDH
